# Overcoming of Microenvironment Protection on Primary Chronic Lymphocytic Leukemia Cells after Treatment with BTK and MDM2 Pharmacological Inhibitors

**DOI:** 10.3390/curroncol28040223

**Published:** 2021-07-01

**Authors:** Erika Rimondi, Elisabetta Melloni, Arianna Romani, Veronica Tisato, Fabio Casciano, Gian Matteo Rigolin, Daniela Milani, Claudio Celeghini, Giorgio Zauli, Paola Secchiero, Rebecca Voltan

**Affiliations:** 1 Department of Translational Medicine and LTTA Centre, University of Ferrara, 44121 Ferrara, Italy; erika.rimondi@unife.it (E.R.); arianna.romani@unife.it (A.R.); veronica.tisato@unife.it (V.T.); fabio.casciano@unife.it (F.C.); paola.secchiero@unife.it (P.S.); rebecca.voltan@unife.it (R.V.); 2 Section of Hematology, Department of Medical Sciences, University of Ferrara, 44122 Ferrara, Italy; gianmatteo.rigolin@unife.it; 3 Department of Translational Medicine, University of Ferrara, 44121 Ferrara, Italy; daniela.milani@unife.it (D.M.); claudio.celeghini@unife.it (C.C.); giorgio.zauli@unife.it (G.Z.)

**Keywords:** leukemia, MDM2 inhibitor, BTK inhibitor, p53, apoptosis

## Abstract

In B-chronic lymphocytic leukemia (B-CLL), the interaction between leukemic cells and the microenvironment promotes tumor cell survival. The Bruton’s tyrosine kinase (BTK) inhibitor ibrutinib is one of the first-in-class molecules for the treatment of B-CLL patients; however, the emerging mechanisms of resistance to ibrutinib call for new therapeutic strategies. The purpose of the current study was to investigate the ability of ibrutinib plus the MDM2-inhibitor nutlin-3 to counteract the tumor microenvironment protective effect. We observed that primary B-CLL cells cultivated in microenvironment mimicking conditions were protected from apoptosis by the up-regulation of c-MYC and of p53. In the same setting, combined treatments with ibrutinib plus nutlin-3 led to significantly higher levels of apoptosis compared to the single treatments, counteracting the c-MYC up-regulation. Moreover, the combination induced high p53 levels and a significant dissipation of the mitochondrial membrane potential, together with BAX cleavage in the more active p18 form and phospho-BAD down-regulation, that are key components of the mitochondrial apoptotic pathway, enhancing the apoptosis level. Our findings propose a new therapeutic strategy to overcome the tumor microenvironment protection involved in B-CLL resistance to drugs, with possible clinical implications also for other hematologic and solid tumors for which ibrutinib is considered a therapeutic option.

## 1. Introduction

The existence of a tumor microenvironment that allows tumor cells to prevent apoptosis or evade immune system control is a crucial feature of cancer, and it is particularly significant for B-chronic lymphocytic leukemia (B-CLL). In this pathology, the bone marrow, lymph nodes, and spleen offer niches of protection for CLL cells, facilitating homing, survival, and the proliferation of leukemia cells through different modalities of interaction with tissue-residing cells [1]. The crosstalk between CLL cells and bystander cells involves receptor–ligand bindings, release of soluble signals (e.g., chemokines) and the engagement of adhesion molecules on cell membrane surfaces, leading to several events regulating cell cycle, apoptosis, metabolism, proliferation, and migration of leukemic cells [2,3]. Overall, this condition results in a protective milieu that offers to CLL cells a protection from both the natural immune response as well as the pharmacological treatments.

In microenvironment mimicking conditions, based on co-culture with fibroblasts or mesenchymal stromal cells and/or stimulation with soluble molecules, such as CD40L and IL4 associated to CpG-oligodeoxynucleotides (CpG-ODNs), several authors have demonstrated the activation of survival pathways, including the B-cell receptor (BCR) pathway, leading to the up-regulation of the key proteins MCL-1 and BCL-XL [4,5,6,7]. In this context, our group preliminarily observed the activation of NOTCH1 and C-MYC pathways in p53 wild-type B-CLL primary cells grown in co-culture with stromal cells [8].

Idelalisib and ibrutinib are being used with success as innovative molecules able to disrupt the microenvironment protection through the inhibition of BCR associated kinases. In particular, ibrutinib is the first-in-class inhibitor of Bruton’s tyrosine kinase (BTK) that is now in clinical use for treatment of B-CLL patients as a category 1 agent in the relapsed/refractory disease [9,10,11]. Moreover, trials have been carried out to investigate its efficacy and safety in the context of treatment-naïve patients [10]. Nonetheless, as seen for other molecules, mechanisms of resistance to ibrutinib are emerging, and, in parallel to the investigation of the safety and efficacy of second-generation BTK inhibitors, novel combination strategies involving ibrutinib and CD20 antibodies, ibrutinib and chemo-immunotherapy, or ibrutinib and venetoclax are under clinical evaluation [12,13,14,15].

In this scenario, we previously demonstrated that the combination of ibrutinib with MDM2-inhibitors synergized in promoting apoptosis in B-CLL cell lines carrying wild-type p53 as well as 17p13 deletion and/or TP53 mutations [16], and that ibrutinib combined with gamma-secretase inhibitors exhibited enhanced cytotoxicity that was coupled with the down-regulation of the c-MYC oncogene [8]. It is known that MDM2-inhibitors (such as nutlin-3) can also down-regulate c-MYC levels following the activation of the p53 pathway [17]. Moreover, it has been revealed that in chronic myeloid leukemia (CML) models, the genetic deregulation driving the disease affects a panel of genes regulated by the oncogenes c-MYC and p53, indicating a tumor-related crosstalk between the two pathways, both controlling survival, differentiation, and apoptosis [18]. In addition, the same authors showed that concomitant pharmacological perturbation of p53 and c-MYC was able to eliminate leukemic stem cells in murine models. Altogether, these observations reinforced our interest in studying these two pathways and the aim of the current study was to investigate the role of the combination ibrutinib plus nutlin-3 in contrasting the B-CLL disease by using a cohort of in vitro microenvironment-activated primary B-CLL samples.

## 2. Materials and Methods

### 2.1. Patients

To assess the preclinical effects of the pharmacologic treatments of BTK and MDM2 inhibitors, blood samples were collected from 26 B-CLL affected patients following full informed consent and after approval of the local Ethical Committee, in accordance with the requirements of the Declaration of Helsinki and the University-Hospital of Ferrara guidelines. Clinical data (CD38 surface expression, IgHV status and cytogenetic abnormalities) of each patient were abstracted from medical records and are showed in Table 1. All patients had been without prior therapy for at least three weeks before blood collection.

CD19^+^ lymphocyte population was purified by the negative depletion of T and NK lymphocytes, granulocytes and monocytes (MACS MicroBeads, Miltenyi Biotech, Auburn, CA, USA) from peripheral blood mononuclear cells (PBMC), isolated by using lymphocyte cell separation medium (Cedarlane, Hornby, ON, USA). The enrichment provided CD19^+^ populations with a purity >95%, evaluated by flow cytometry. Primary lymphocytes, freshly isolated or cryopreserved, were cultured in RPMI-1640 medium supplemented with 10% FBS, L-glutamine, and penicillin/streptomycin (all from Gibco, Grand Island, NY, USA).

### 2.2. Next Generation Sequencing (NGS)

Genomic DNA was extracted from 2.5 × 10^6^ purified CD19^+^ lymphocytes by using the QIAamp DNA kit (Qiagen, Hilden, Germany). An Ion Ampliseq panel was designed targeting all coding sequences and the exon–intron boundary regions up to 25-bp of the splicing junctions of the gene TP53. For DNA library construction, two primer pools were designed by Ion AmpliSeq™ Designer v4.2 (Life Technologies, Foster City, CA, USA). Targeted NGS was performed by the Microarray Facility of the University of Ferrara using Ion Torrent PGM (Life technologies, Foster City, CA, USA), as previously described [19]. Sequencing results were compared to the human reference genome (GRCh37). Data analysis and variants identification were carried out by the Torrent Suite 3.4 and Variant Caller plugin 3.4.4 software (Life technologies). All data are available at the public archive European Variation Archive (EVA) with the accession number PRJEB34654.

### 2.3. Pharmacological Treatments and Evaluation of Cell Viability and Apoptosis

B-CLL primary cells were seeded at 2 × 10^6^ cells/mL and either co-cultured with a confluent culture of stromal cells (NIH/3T3, ACC 59, DMSZ, Braunschweig, Germany), or stimulated with 1 μg/mL CD40L (Peprotech, London, UK) and 1.5 μg/mL CpG-ODN (ODN2006; Invivogen, San Diego, CA, USA), or cultured unstimulated as a control in complete RPMI-1640 medium (Gibco).

For pharmacological treatments, primary B-CLL cells were exposed to ibrutinib (PCI-32765; Selleckchem, Houston, TX, USA), used as single therapy or in combination with nutlin-3 (Cayman Chemical, Ann Arbor, MI, USA), both suspended following manufacturer’s instruction and used at the previously selected concentration of 10 μM [16] and as previously described by other authors [20,21,22]. Cells exposed only to the vehicle were used as a reference negative control (untreated). To evaluate the cytotoxic effects of the drugs, primary B-CLL cells were harvested and cell viability as well as apoptosis were analyzed at different time points after treatments (24 and 48 h for viability, 48 h for apoptosis). The harvest procedure for co-cultures was performed by gently washing off the B-CLL cells and leaving the adherent stromal cell layer intact on the plates. In order to assess CLL viability, cells were examined by Trypan blue dye exclusion. The percentage of apoptosis was determined by flow cytometry (FACSCalibur; BD Biosciences, San Josè, CA, USA) following Annexin V-FITC/propidium iodide (PI) double staining (Beckman Coulter Inc., Brea, CA, USA), as previously described [23]. Apoptosis data analysis was performed using the FloJo software (Tree Star, Ashland, OR, USA).

### 2.4. Mitochondrial Activity Assessment

In order to evaluate the mitochondrial activity, leukemic cells were treated with ibrutinib, nutlin-3 or their combination as described above, and then labeled with MitoTracker^®^ Red CMXRos (Molecular Probes, Inc., Eugene, OR, USA) that stains the mitochondria of live cells depending on the mitochondrial membrane potential. Briefly, 24 h post treatment, cells were incubated for 30 min at 37 °C with 100 nM MitoTracker^®^ Red CMXRos, washed with PBS (Gibco) and analyzed by flow cytometry (FACSCalibur, BD Biosciences, San Josè, CA, USA). Data were analyzed with the FlowJo software 9.9.6 (Tree Star).

### 2.5. RNA and Protein Analyses

In order to evaluate how ibrutinib and nutlin-3 affect gene and protein expression of several components of p53 and BTK pathways, and to understand possible common or interrelated molecular targets, leukemic cells were harvested 24 h after treatments and processed for RNA and protein extraction as follows.

Total RNA was purified from lymphocytes by using the QIAGEN miRNeasy Mini kit (QIAGEN, Hilden, Germany), according to the supplier’s instructions. Total RNA (300 ng) was retro-transcribed and amplified by using the Express One-Step Superscript qRT-PCR Kit universal. Analysis of human c-MYC, TP53, MDM2 and CDKN1A gene expression was performed with specific primers sets (TaqMan Gene Expression Assays; c-MYC assay: Hs00153408_m1; TP53 assay: Hs01034249_m1; MDM2 assay: Hs00242813_m1; CDKN1A assay: Hs00355782_m1). Samples were run in duplicate by using the QuantStudio^®^ 3 Real-Time PCR System, and expression values were normalized to the housekeeping gene POLR2A (assay Hs00172187_m1). These values were used to perform statistical analyses. All material was from Thermo Fisher Scientific (Rockford, IL, USA). 

For Western blotting analysis, cells were processed as previously described [24] and equal amounts of protein were loaded for each sample. The following antibodies were used for immunoblotting onto nitrocellulose filters: anti c-MYC, anti-phospho p53-Ser15, anti-phospho p53-Ser392, BAD, phospho BAD-Ser136 and p14ARF from Cell Signaling (Danvers, MA, USA); anti-p53, anti-MDM2, anti-p21, anti-PUMA, anti-procaspase 9 and anti-procaspase 3 from Santa Cruz Biotechnology (Santa Cruz, CA, USA); anti-BAX and anti-Akt from BD (BD Biosciences); phospho Akt-Ser473 from Merck Millipore (KGaA, Darmstadt, Germany) and anti β-actin, from Sigma-Aldrich (Merck KGaA). After incubation with secondary antibodies (anti- mouse or -rabbit IgG HRP-conjugated; Sigma-Aldrich), specific band detection was performed with the ECL Lightning kit (Perkin Elmer, Waltham, MA, USA). Images’ acquisition and analysis was performed using the ImageQuant™ LAS 4000 imager and TL software (GE Healthcare, Buckinghamshire, UK).

### 2.6. Statistical Analysis

Statistical analysis of at least three independent experiments were expressed as median or mean ± standard deviation (SD). Normal distribution was tested by a Kolmogorov–Smirnov test. For the not-normally-distributed variable, base-10 logarithm transformation was performed before statistical analysis. The results were evaluated by unpaired Student’s *t*-test or unpaired one-way ANOVA with a Bonferroni post hoc test (for multiple comparison). Probability value *p* < 0.05 was considered statistically significant. Analyses were performed by using GraphPad Prism, version 8.0 (GraphPad Software, La Jolla, CA, USA).

## 3. Results

### 3.1. Microenvironment-Activated B-CLL Cells Display Up-Regulation of c-MYC and p53

Since the sensitivity of B-CLL to treatments is heavily reduced by the microenvironment within the lymphoid organs, in the present study we have analyzed primary cells isolated from 26 B-CLL affected patients (Table 1) co-cultured in the presence of stroma or stimulated with CpG-ODN and CD40L, to mimic the lymph node environment in vitro.

**Figure 1:**
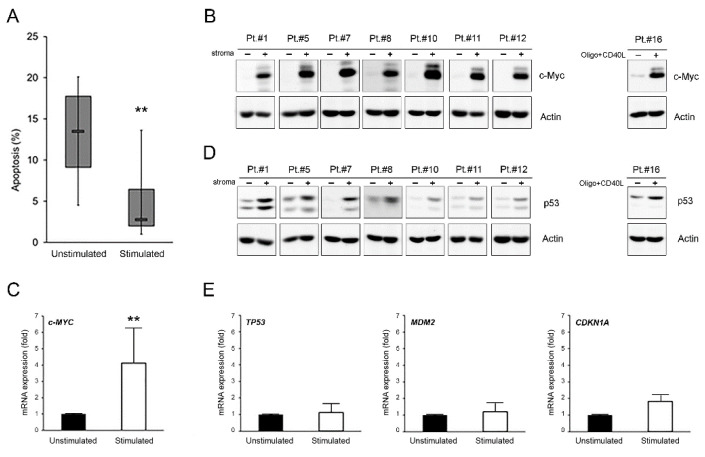
c-MYC and p53 induction in microenvironment-activated B-CLL cells. Patients’ derived B-CLL cells were stimulated (with stroma or CpG-ODN and CD40L) for 24–48 h or grown unstimulated in suspension as control. In (**A**), baseline apoptosis level, indicated as percentage of Annexin V/PI double positive cells, was comparatively assessed in B-CLL cells unstimulated and stimulated with stroma at 48 h. Horizontal bars are median, upper and lower edges of box are 75th and 25th percentiles, lines extending from box are 10th and 90th percentiles. The two asterisks indicate *p* < 0.01 with respect to unstimulated cells (Student’s *t*-test). Results are reported as mean ± SD of four independent experiments. In (**B**,**D**), Western blotting analyses of c-MYC and p53 protein levels are shown at 24 h for representative B-CLL patients. β-actin levels are shown as loading control. In (**C**,**E**), levels of c-MYC, TP53, MDM2, and CDKN1A mRNA were analyzed by qRT-PCR at 24 h in B-CLL cells unstimulated and stimulated with stroma and expressed as fold of modulation with respect to the B-CLL cultures grown in suspension without stimulation (unstimulated, set at 1). Results are reported as mean ± SD of three independent experiments. The two asterisks (**) indicate *p* < 0.01 with respect to cells grown untreated in suspension (unstimulated) (Student’s *t*-test).

As expected, this setting offered significant (*p* < 0.01) protection from spontaneous apoptosis to all B-CLL samples (Figure 1A). To evaluate the possible involvement of c-MYC and p53 in the survival of microenvironment-activated B-CLL cells, we firstly analyzed their expression levels under these baseline conditions. As expected, we documented a significant up-regulation of the proto-oncogene c-MYC both at protein and mRNA levels (*p* < 0.05) in all samples analyzed (Figure 1B,C and Figure S1). At the same time, we observed that the c-MYC up-regulation was accompanied by a p53 protein level increase (Figure 1D and Figure S1), as previously also seen by Althubiti et al. [25], but not by the up-regulation of the expression of the TP53 gene itself or its canonical targets CDKN1A and MDM2 (Figure 1E).

### 3.2. Ibrutinib and Nutlin-3 Combination Efficiently Kills Microenvironment-Activated Leukemic Cells

**Figure 2:**
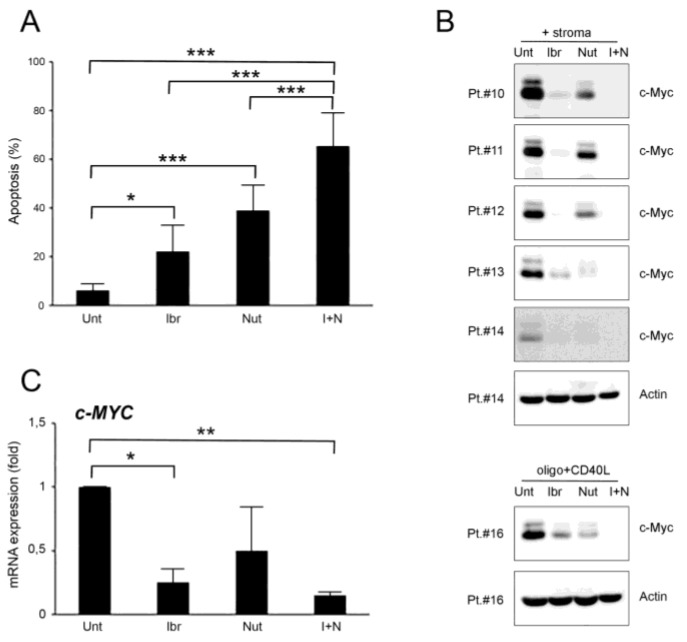
Ibrutinib and nutlin-3 combined treatment increases cytotoxicity in B-CLL activated cells. Patients’ derived B-CLL cells were grown in vitro with stroma or with CpG-ODN and CD40L and exposed to ibrutinib (10 µM), nutlin-3 (10 µM), ibrutinib+nutlin-3 (10 µM), or left untreated as a control, for 24–48 h. In (**A**), apoptosis level in response to treatments, calculated as percentage of Annexin V/PI double positive cells, was comparatively assessed after 48 h of treatment in cells stimulated with stroma. Results are reported as mean ± SD of seven independent experiments. In (**B**), Western blotting analyses of c-MYC protein levels 24 h post-treatment are shown for representative B-CLL patients. For illustrative purposes the untreated bands are re-used from Figure 1, and, for clarity, β-actin is shown as loading control for two patients. In (**C**), levels of *c-MYC* mRNA were analyzed by qRT-PCR at 24 h in cells stimulated with stroma and are expressed as fold of modulation with respect to untreated B-CLL cultures (set at 1). Results are reported as mean ± SD of three independent experiments. Asterisks indicate: *, *p* < 0.05; **, *p* < 0.01; ***, *p* < 0.001 (ANOVA followed by Bonferroni post-hoc test or Student’s *t*-test).

To investigate if ibrutinib and nutlin-3 were able to target microenvironment-protected leukemia cells, primary B-CLL cells co-cultured with stroma or stimulated with CpG-ODN and CD40L were treated with the two drugs used alone or in pharmacological combination. The cytotoxicity analysis showed a significant (*p* < 0.001) rise of apoptotic level in samples treated with the combination, respective to treatment with ibrutinib or nutlin-3 as monotherapy, indicating that the combination was efficient in overcoming the protection offered by the microenvironment to leukemia cells (Figure 2A). Of note, during these experiments, we never observed interference of ibrutinib and/or nutlin-3 on stroma viability.

At the molecular level, the results showed that both ibrutinib and nutlin-3 were able to counteract c-MYC up-regulation even if they target different pathways, both at the protein and mRNA level, and that this effect was significantly enhanced by the combined use of the two drugs (Figure 2B,C and Figure S1). Moreover, the c-MYC down-modulation trend induced by the single treatments was not directly correlated with the relative apoptotic percentages as shown in Figure 2A, suggesting a cooperation of the two drugs also in other pathways or with other molecular mechanisms that converge on cellular death when the drugs were used together. Interestingly, the down-regulation of c-MYC mediated by ibrutinib and the ibrutinib plus nutlin-3 combination was independent of the B-CLL patients’ clinical characteristics (Figure 2B,C and Table 1).

### 3.3. Effects of Ibrutinib and Nutlin-3 Combination on p53 Pathway

**Figure 3:**
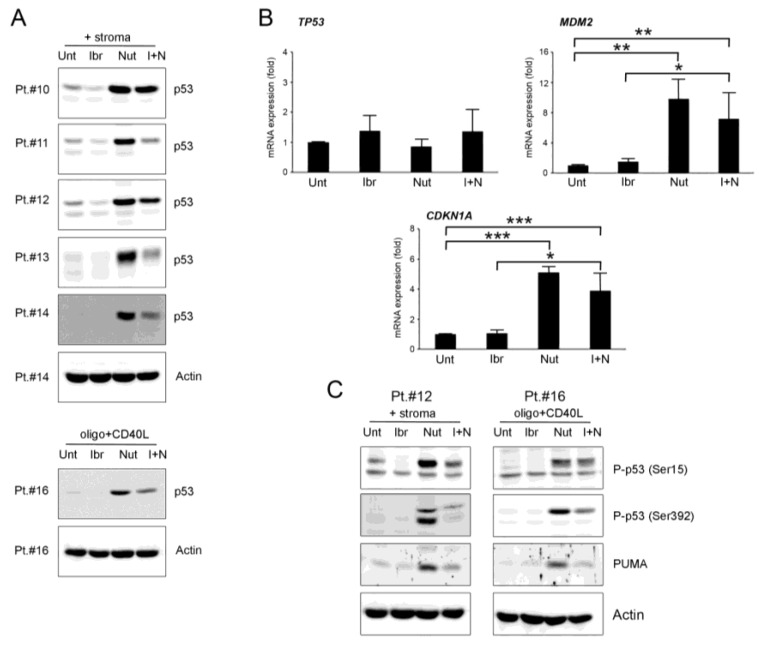
Ibrutinib down-regulates p53 pathway. Patients’ derived B-CLL cells were grown in vitro with stroma or with CpG-ODN and CD40L and exposed to ibrutinib (10µM), nutlin-3 (10 µM), ibrutinib+nutlin-3 (10 µM) or left untreated, for 24–48 h. In (**A**), Western blotting analyses of p53 protein levels after 24 h of treatment are shown for representative B-CLL patients. For illustrative purposes the untreated bands are re-used from Figure 1, and, for clarity, β-actin is shown as loading control for two patients. In (**B**), *TP53, MDM2* and *CDKN1A* mRNA levels were analyzed by qRT-PCR at 24 h in cells stimulated with stroma and are expressed as fold of modulation with respect to the untreated B-CLL cultures (set at 1). Results are reported as mean ± SD of four independent experiments. Statistical analyses were performed by ANOVA followed by Bonferroni post-hoc test. Asterisks indicate: *, *p* < 0.05 **, *p* < 0.01; ***, *p* < 0.001. In (**C**), Western blotting analyses of p53 phosphorylation in ser15 and ser392 and PUMA protein levels after 24 h of treatment are shown for representative B-CLL patients. β-actin levels are displayed as loading control.

In parallel to the down-regulation of c-MYC, we observed that ibrutinib was able to also down-regulate p53 protein level, induced by stroma or CpG-ODN plus CD40L stimulation, when used as a single treatment or in combination with nutlin-3 (Figure 3A and Figure S1). Indeed, ibrutinib was able to modulate the p53 protein to lower levels even when the p53 protein was induced to high levels because of the inhibition of MDM2 mediated by nutlin-3 (Figure 3A). In both cases, the reduction of the p53 protein was not accompanied by a reduction of TP53 gene expression, supporting a post-transcription/translation modulation of the p53 protein (Figure 3B). At the mRNA level, the down-regulation of p53 induced by ibrutinib plus nutlin-3 treatment compared to nutlin-3 alone was mirrored by a reduction of the p53-target genes MDM2 and CDKN1A expression (*p* < 0.05) (Figure 3B). 

Considering that the down-modulation of p53 did not match the increased apoptotic levels of cells exposed to the combination, we studied if the p53 down-modulation reflected a different activation of the protein through altered phosphorylation. Therefore, we have analyzed ser15 and ser392 p53 phosphorylation since they are critical for the transactivation and mitochondrial translocation of p53, respectively. The results showed that both ser15 and ser392 phosphorylation were down-regulated by ibrutinib used as monotherapy or in combination with nutlin-3 (Figure 3C and Figure S1), with a trend similar to the down-regulation observed for the p53 total protein. The phospho-p53 levels from cells treated with nutlin-3 alone were also perfectly in line with the total p53 level. These results indicated that the cytotoxic effects induced by the combinatorial treatment were not related with the activation of p53 protein. 

Next, we analyzed the levels of the relevant pro-apoptotic p53-target protein PUMA. The results evidenced that PUMA protein level was enhanced by nutlin-3 treatment, confirming that the apoptosis induced by nutlin-3 alone was guided by PUMA, as previously observed by Valente et al. [26], and that it was down-regulated by the combination with ibrutinib, showing that PUMA had low relevance in enhancing the apoptosis level seen by the combination of the two drugs (Figure 3C).

### 3.4. Ibrutinib Plus Nutlin-3 Combination Mediates Mitochondria-Dependent Apoptosis

To further investigate the molecular mechanism that, despite the p53 down-regulation by ibrutinib, was involved in the enhanced cytotoxic effect induced by the simultaneous treatment with nutlin-3, we analyzed some relevant events of the apoptotic cascade.

**Figure 4:**
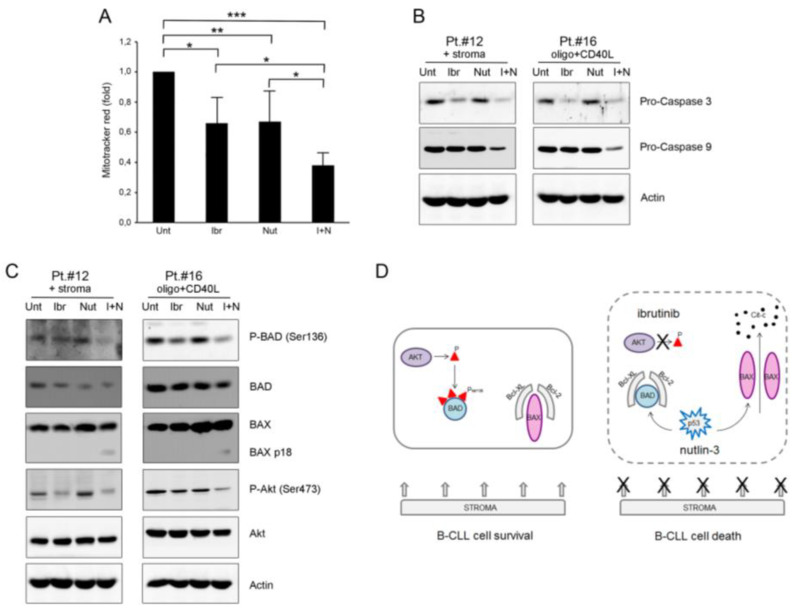
Ibrutinib plus nutlin-3 combination mediates mitochondria-dependent apoptosis through inhibition of phospho-BAD. Patients’ derived B-CLL cells were grown in vitro with stroma or with CpG-ODN and CD40L and exposed to ibrutinib (10 µM), nutlin-3 (10 µM), ibrutinib+nutlin-3 (10 µM) for 24 h or left untreated as control. In (**A**), mitochondrial activity, evaluated using MitoTracker^®^ Red staining on B-CLL cells stimulated with CpG-ODN and CD40L, was analyzed by flow cytometry and expressed as fold of modulation with respect to the MFI value of the untreated B-CLL cultures set at 1. Results are reported as mean ± SD of five independent experiments. Statistical analysis was performed by ANOVA followed by Bonferroni post-hoc test. Asterisks indicate: *, *p* < 0.05; **, *p* < 0.01; ***, *p* < 0.001. In (**B**), Western blotting analyses of pro-caspase 9 and pro-caspase 3 protein levels are shown. In C, Western blotting analyses of BAD phosphorylation in ser136, total BAD, BAX, Akt phosphorylation in ser473 and total Akt protein levels are shown. In (**B**,**C**), results are shown for representative B-CLL patients and β-actin levels are shown as loading control. In (**D**), schematic representation of early molecular events that drive the mitochondria-dependent apoptosis following the combination treatment ibrutinib plus nutlin-3.

As the first step, we investigated the involvement of the mitochondria by the MitoTracker^®^ Red staining assay. Results showed a significant (*p* < 0.05) dissipation of the mitochondrial membrane potential in cells treated with the combination ibrutinib plus nutlin-3 (Figure 4A), suggesting the activation of the intrinsic mitochondria-dependent apoptotic pathway. This result was supported by the progressive cleavage of pro-caspase 9 and pro-caspase 3 also involved in the “intrinsic” mitochondrial pathway (Figure 4B and Figure S1).

We then analyzed some early molecular events that drive the mitochondria-dependent apoptosis, focusing on the pro-apoptotic proteins BAD as a sensitizer and BAX as the active molecule triggering apoptosis. Interestingly, analysis of phosphorylated BAD showed evident down-modulation of ser136-phosphorylation following the pharmacological combination ibrutinib plus nutlin-3 (Figure 4C and Figure S1). This result might explain, at least partially, the high apoptosis percentage observed with the combination. Notably, Akt, a protein kinase involved in the BCR signaling and in BAD ser136-phosphorylation, was also inhibited by ibrutinib and by the combination of ibrutinib plus nutlin-3 (Figure 4C).

Next, we analyzed BAX. Results showed a high background expression of the BAX protein in all samples analyzed (Figure 4C). Interestingly, only the combination induced a supplemental BAX band, indicating full-length p21 BAX cleavage to the more active short p18 form (that is usually visible only in the mitochondrial-enriched lysates). This result supported the involvement of the mitochondria in the cytotoxicity induced by the combination (schematically represented in Figure 4D).

## 4. Discussion

It is well-known that microenvironment has a protective role for leukemia cells that can evade immune surveillance, proliferate, and resist pharmacological treatments thanks to the activation of survival pathways [1,2,3,4,5,6,7,8]. In this scenario, we initially observed apoptosis protection of CLL cells associated with the up-regulation of c-MYC and p53 proteins. While the up-regulation of c-MYC correlated well with literature data reporting high expression of this protein in highly proliferative tumoral cells, and with our preliminary observations on leukemia cells [8,27], the up-regulation of p53 seemed to be in contrast with the role of p53 in the apoptosis induction. We also observed that the increased level of the p53 protein was not followed by the induction of relevant p53 targets, including MDM2. These data could be explained by a stabilizing role of the BCR signaling, whose activation was confirmed by the c-MYC up-regulation, on p53 protein and was in line with data obtained by other authors in previous studies [25,28].

The collaboration of the two drugs, ibrutinib and nutlin-3, on cMYC down-regulation is particularly interesting and is supported by previous observations indicating that ibrutinib acts in this pathway indirectly through the inhibition of the BTK cascade [29], while the mechanism of the action of nutlin-3 is mediated by miR34a [17]. This could have important implications because of the roles of c-MYC in cell metabolism and cancer [27], revealing new therapeutic opportunities so far not investigated for both ibrutinib and nutlin-3 as alternative molecules to canonical c-MYC inhibitors. Moreover, the combined use of ibrutinib and nutlin-3, both able to reduce the levels of c-MYC in independent manner, could represent a new pharmacological strategy to escape the resistance to inhibitors of BCR signaling in B cell non-Hodgkin lymphoma (B-NHL) models expressing elevated levels of c-MYC [30].

The effect of the two drugs on p53 is more complex. While nutlin-3, as expected, promoted the up-regulation of the p53 protein, ibrutinib worked apparently in the opposite direction, promoting its down-regulation and acting as antagonist of nutlin-3 on the p53 pathway. Further, this observation can be partially explained by the discovery made by Althubiti et al. showing that BTK can directly phosphorylate p53 and stabilize it [25], so when ibrutinib inhibits the kinase activity of BTK, it can reduce the stability of p53. It is not clear, however, how ibrutinib can down-regulate p53 induced by nutlin-3, considering that nutlin-3 effects are, to our knowledge, BTK-independent. Moreover, we observed that ibrutinib reduced the p53 total level without altering the physiological function of the protein [31,32].

How the two drugs functioned together in amplifying the apoptosis of CLL primary cells appeared clear in mitochondrial examination. Their cooperation was strengthened by the down-regulation of Akt kinase that reduced the phosphorylation of ser136-BAD [33]. De-phosphorylated BAD is the only status of BAD that allows apoptosis. Of note, BAD is a target gene of p53 transactivation activity and p53 itself can form a protein complex with dephosphorylated BAD, thereby participating in the activation of BAD to a pro-apoptotic molecule [34]. In fact, only the de-phosphorylated BAD allows apoptosis, because it forms heterodimers with the anti-apoptotic molecules Bcl-2 and Bcl-Xl, inactivating them and leaving BAX free to oligomerize and to form mitochondrial membrane pores. Although full-length p21 BAX can initiate apoptosis through the mitochondrial pathway, p18 BAX is more potent than p21 BAX in disrupting mitochondrial integrity and inducing apoptosis. Therefore, the cleavage of BAX to p18 BAX, operated by calpain, functioned as an amplification step that accelerated the apoptotic events [35].

In conclusion, our results indicate that dual BTK and MDM2 inhibition was able to induce a robust apoptosis of B-CLL cells in a microenvironment protection setting with mitochondria involvement. Our data could have notable clinical implications. In fact, even if ibrutinib therapy alone produces durable remission in the majority of CLL affected patients, the cases of resistance are increasing [9,12,13,36,37], suggesting that it is opportune to find new strategies to defeat CLL using combined approaches. In this respect, recent data have highlighted the use of ibrutinib as a sensitizer for traditional chemotherapeutics, such as paclitaxel, in chemo-resistant tumors [38]. In addition, ibrutinib is becoming an interesting molecule for other BTK-overexpressing cancers, such as colon cancer [39]. On the other hand, recently, nutlin-3 has also been considered for innovative pharmacological combinations in colon and lung cancer therapy [40,41], even if it is necessary to be aware about the potential risk of a low response in patients with mutated p53. Nevertheless, we demonstrated previously in preclinical models that the use of nutlin-3 in combination with ibrutinib, sorafenib or dasatinib was effective on wild-type p53, as well as on p53 mutated/deleted leukemia samples [16,20,42]. Our results, together with the considerations about the broadening effects of ibrutinib and nutlin-3 against other cancer types, suggest that it could be of interest to further investigate their combined effects for new approaches against hematological cancers as well as solid tumors.

## 5. Conclusions

In conclusion, our results demonstrate the effectiveness of the in vitro combinatorial treatment with ibrutinib and nutlin-3 and highlight the importance of continuing to look for new therapeutical strategies able to overcome the tumor microenvironment protection to fight the onset and the development of B-CLL resistance. These observations could also be of high significance for the possible clinical implication in the therapy of other hematologic malignancies and solid tumors.

## Figures and Tables

**Table 1:** Clinical and laboratory characteristics of B-CLL patients at the moment of the in vitro treatments.

Patient Demographics			B-CLL Characterization						
Pt. #	Age	Sex	WBC × 10^3^/Lymphocytes (%)	CD38^+ †^	IgHV status	Cytogenetic Abnormalities *	*TP53* Status (%)	Therapy	
1	76	M	67.1/84.7	pos	mut	del13q	c.380C>T (39.6%), c.920-2A>G (splicing) (26.2%)	R-ibr	
2	58	M	84.3/84.2	neg	unmut	neg	unmut	FCR	
3	85	F	54.8/90.2	pos	mut	del13q, trisomy 12	unmut	steroids	
4	68	M	122/89.5	neg	unmut	del11q	unmut	Ibr	
5	57	F	181/97.8	neg	mut	del13q	unmut	R-ibr	
6	84	F	99/94.4	neg	mut	del13q	unmut	Chl + Pdn	
7	84	M	69.6/88.2	neg	na	neg	unmut	untreated	
8	36	F	197/80.3	pos	unmut	neg	unmut	FCR	
9	66	M	23.9/83.7	neg	unmut	del13q	unmut	Ibr	
10	76	F	na	pos	unmut	del13q	unmut	R-benda	
11	59	M	194.9/90.6	neg	unmut	trisomy 12	unmut	FCR	
12	58	M	24.6/81.3	neg	mut	del13q	unmut	untreated	
13	71	M	73.6/91.5	neg	mut	del13q	unmut	untreated	
14	59	F	52.2/90.2	pos	na	del11q	unmut	FCR	
15	56	M	82.6/94.3	pos	unmut	trisomy 12	unmut	FCR	
16	61	M	49.5/66.5	neg	unmut	trisomy 12	c.G743>A (1%), c.G527>T (11.3%)	ibr	
17	60	M	34/80.1	pos	na	del13q, del11q, trisomy 12	c.G733>A (17.4%)	ibr + ofatum	
18	75	M	10.4/88.3	neg	mut	del13q	unmut	untreaed	
19	69	F	156/92.8	pos	unmut	neg	unmut	R-Benda	
20	61	M	17.2/63.2	neg	na	del13q	unmut	untreated	
21	76	M	168/87.2	neg	mut	del13q, del11q	unmut	untreated	
22	81	M	12.7/33	neg	na	neg	unmut	untreated	
23	50	F	35.1/88.4	neg	na	del13q	unmut	untreated	
24	72	M	33.2/87.3	pos	na	del11q, trisomy 12	na	untreated	
25	73	M	78.4/92.2	neg	mut	neg	unmut	untreated	
26	63	F	40.7/84.1	pos	unmut	del13q	c.G475C (3.6%)	FCR	

List of abbreviations: chl, Chlorambucil; del, deletion; FCR, Fludarabine-Cyclophosphamide-Rituximab; ibr, ibrutinib; IgHV, immunoglobulin heavy chain variable region; mut, mutated; na, not available; neg, negative; Ofatum, ofatumomab; Pdn, Prednisone; R-Benda, Rituximab-Bendamustine; R-ibr, rituximab-ibrutinib; unmut, unmutated; WBC, white blood cells; Pt., patient; #, patient’s number. † Results obtained in cytometric assays, using a CD38 cutoff of 30%. * Cytogenetic abnormalities were evaluated by fluorescence in situ hybridization (FISH) analysis.

## Data Availability

The NGS datasets generated/analyzed for this study can be found in the public archive EVA with the accession number PRJEB34654.

## Supplementary Materials

The following are available online at https://www.mdpi.com/article/10.3390/curroncol28040223/s1, Figure S1: Original Western blots.
