# Supplementary material for: Overcoming of Microenvironment Protection on Primary Chronic Lymphocytic Leukemia Cells after Treatment with BTK and MDM2 Pharmacological Inhibitors
Source: Curr Oncol. 2021 Jul 1;28(4):2439–51. doi: 10.3390/curroncol28040223 (PMC8293193; doi:10.3390/curroncol28040223)

Original blot for Figure 1B-D

c-Myc

Pt.#1

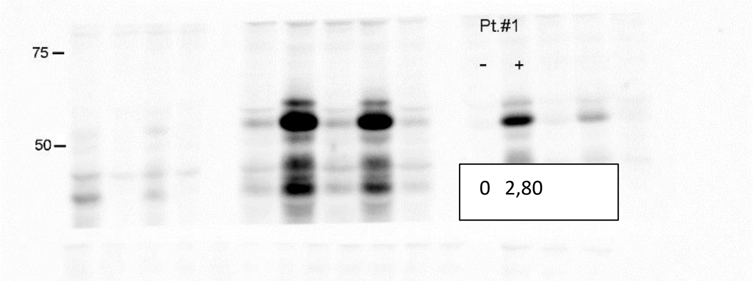

Pt.#5

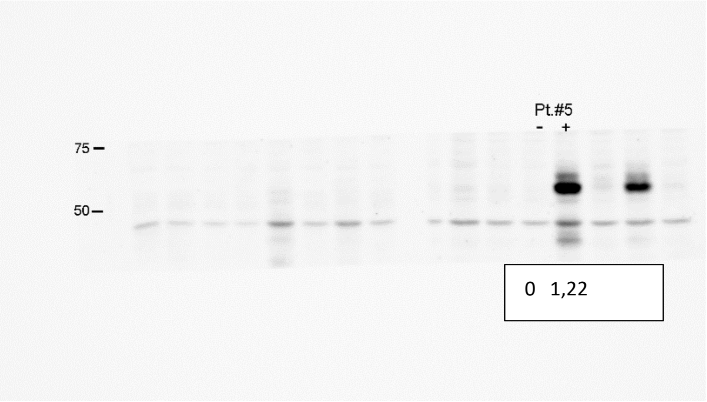

Pt.#7

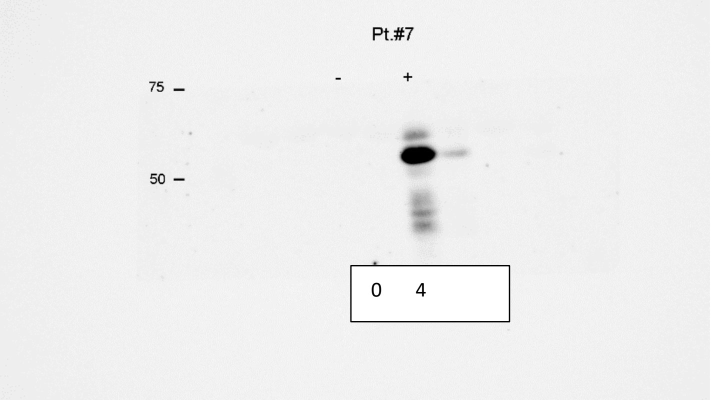

Pt.#8

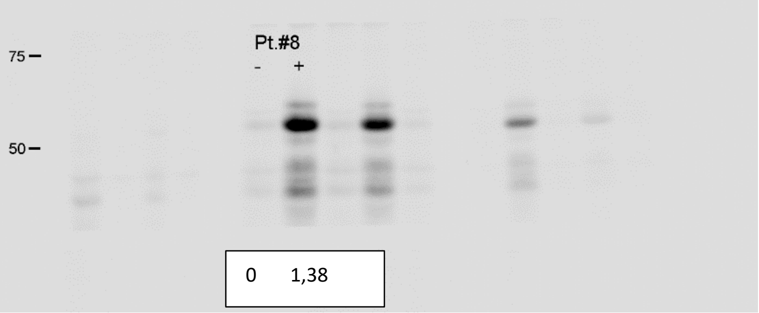

Pt.#10

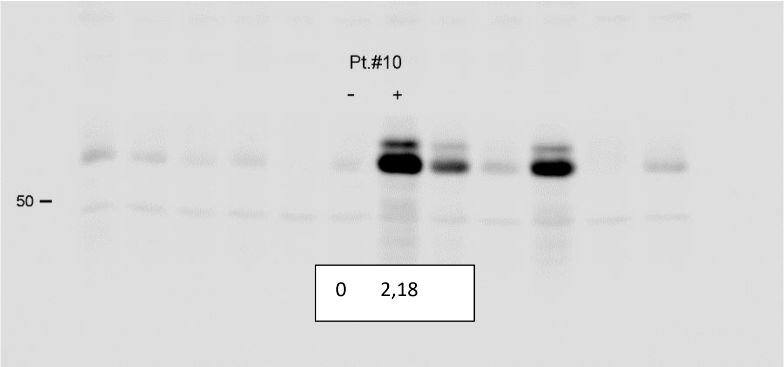

Pt.#11

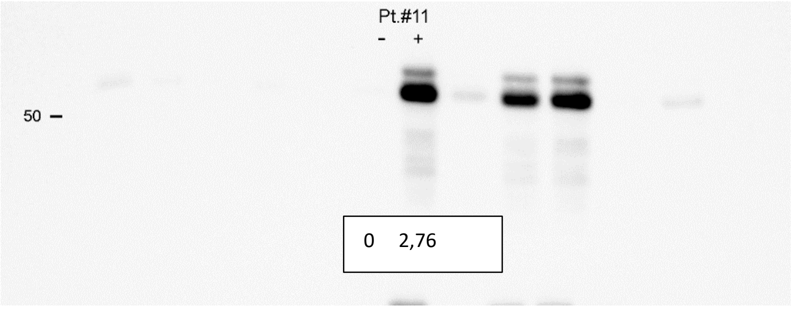

Pt.#12

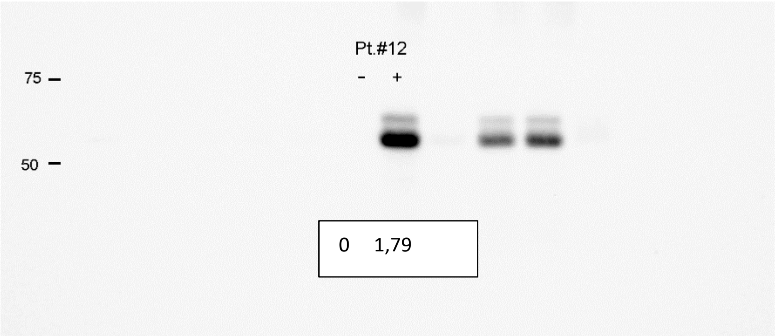

Pt.#16

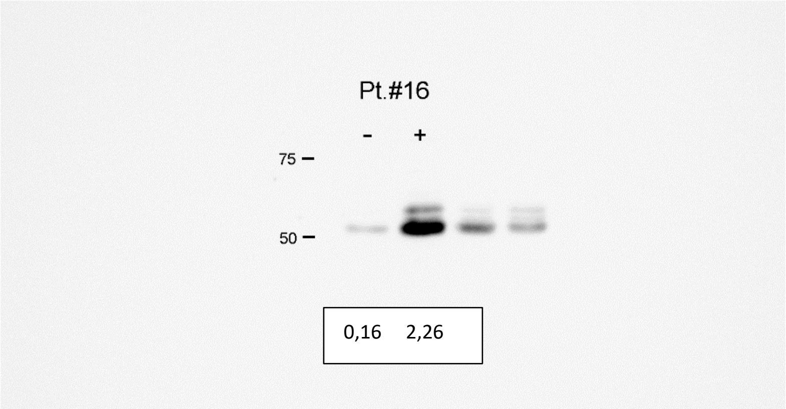

p53

Pt.#1

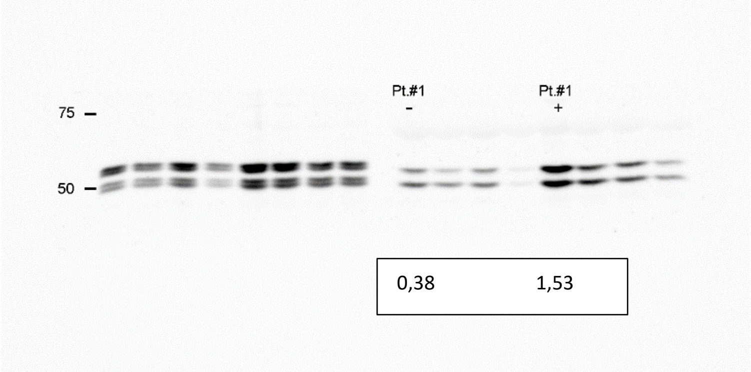

Pt.#5

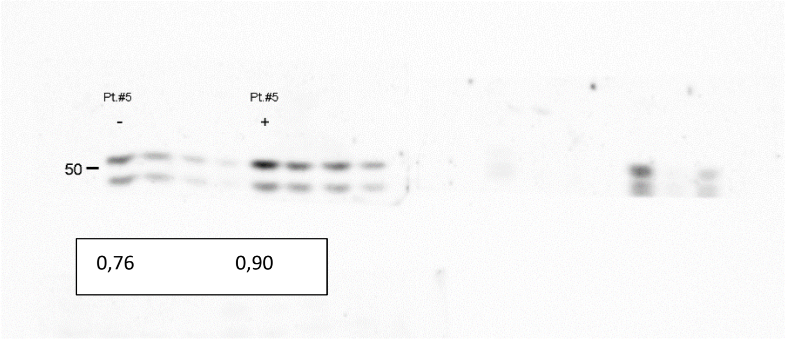

Pt.#8

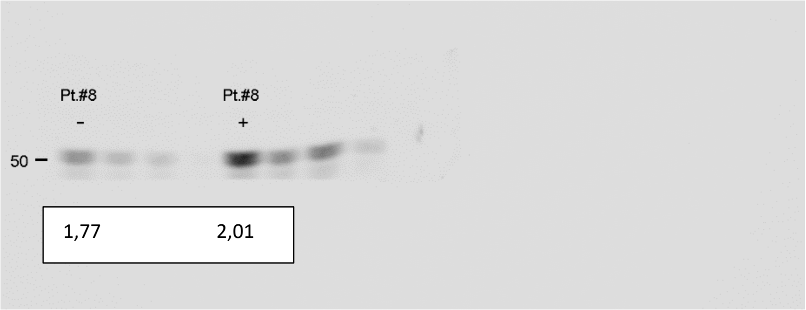

Pt.#10

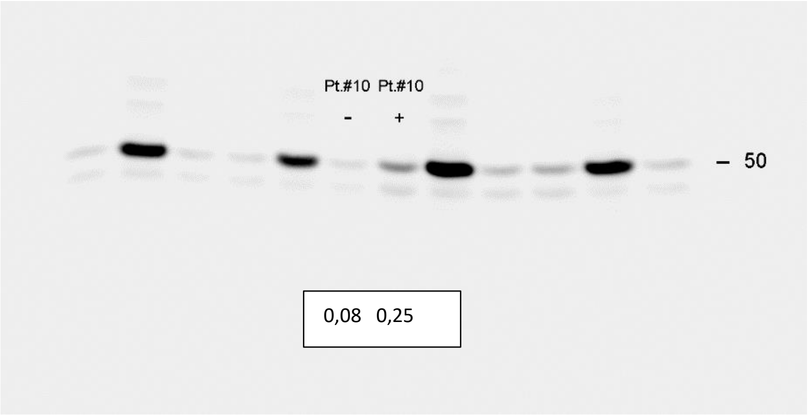

Pt.#11

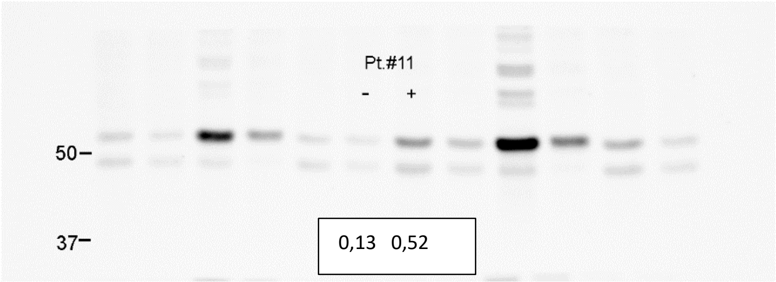

Pt.#12

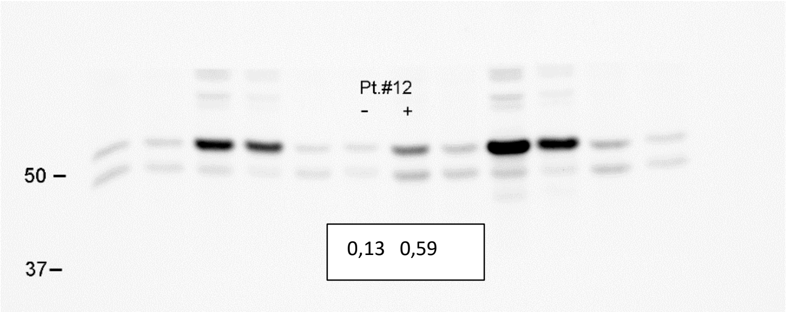

Pt.#16

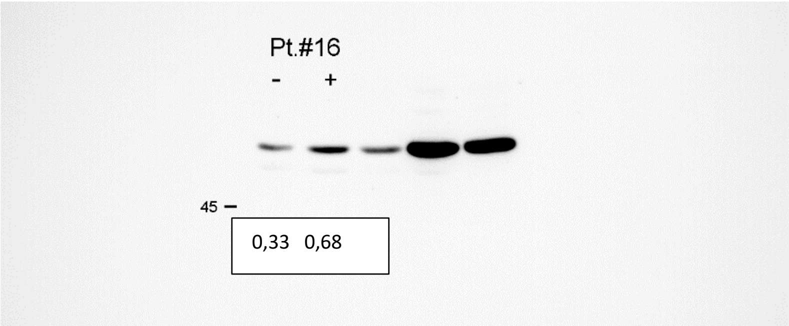

Actin

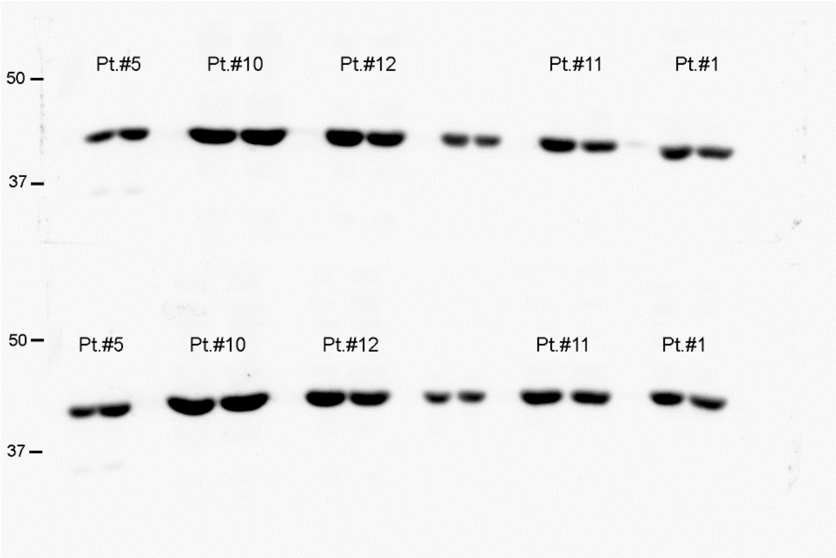

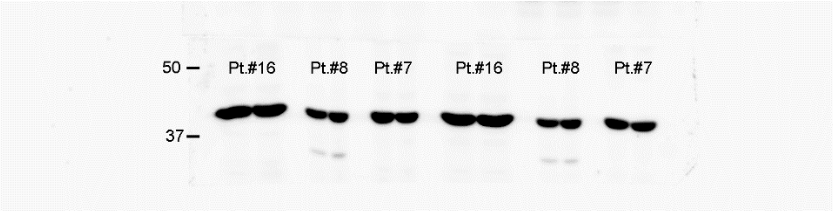

Original blot for Figure 2B

c-Myc

Pt.#10

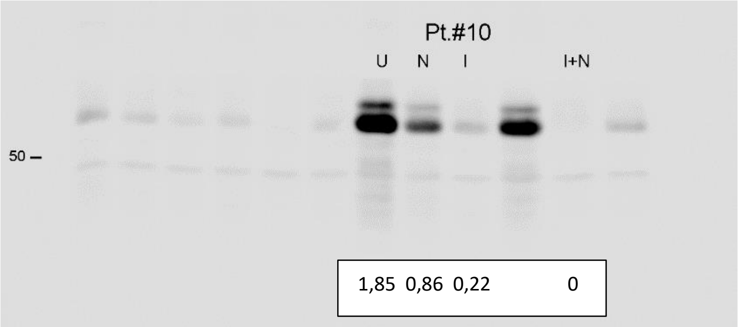

Pt.#11

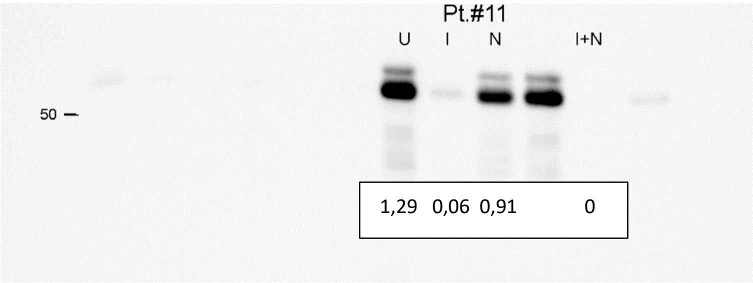

Pt.#12

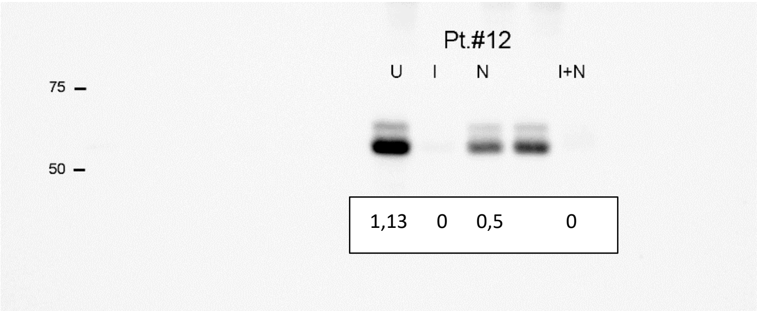

Pt.#13

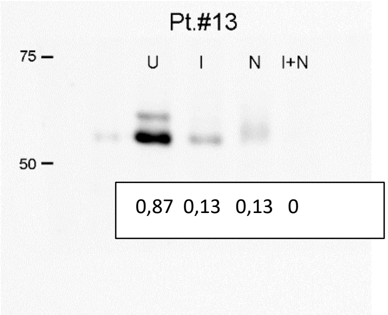

Pt.#14

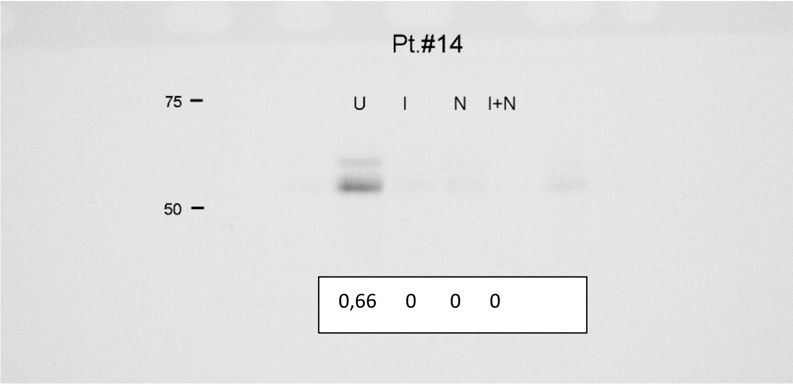

Actin

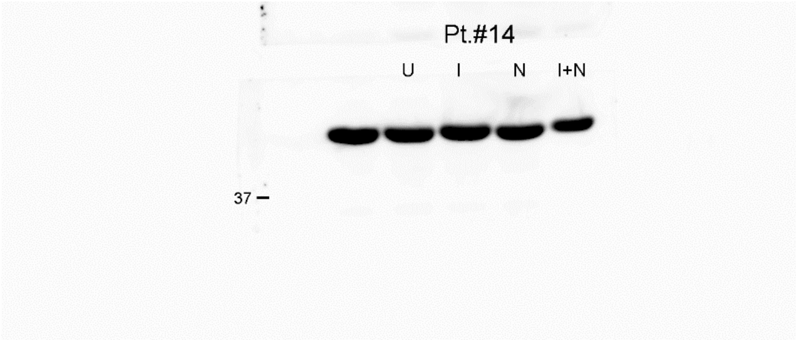

Pt.#16 c-Myc

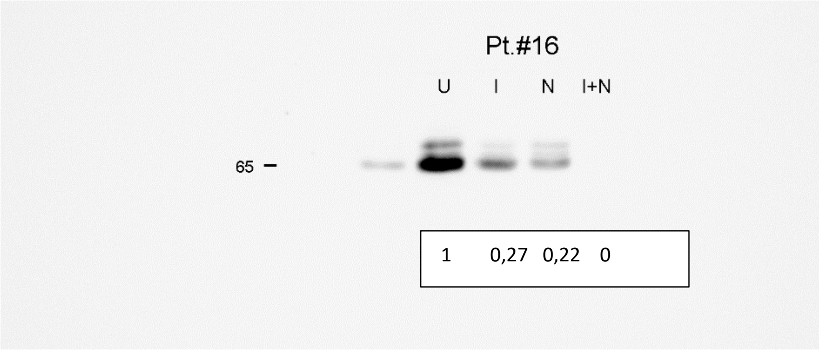

Actin

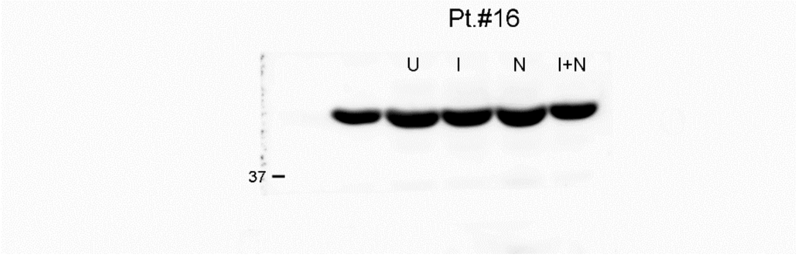

Original blot for Figure 3A

P53

Pt.#10

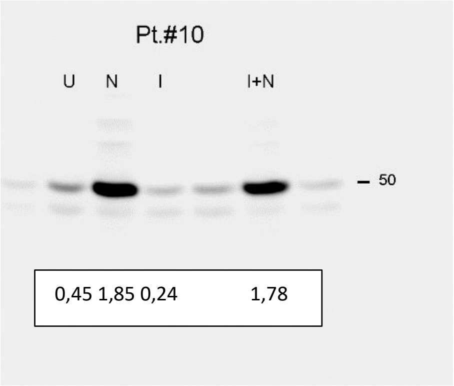

Pt.#11

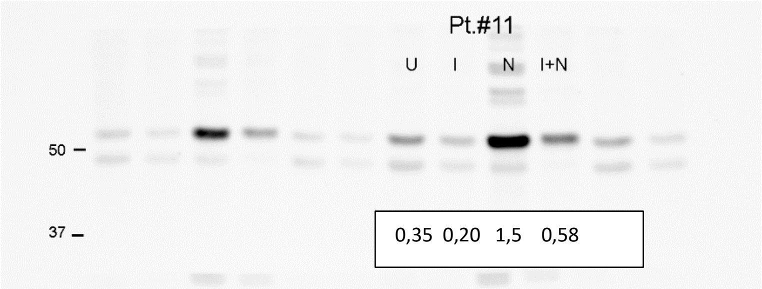

Pt.#12

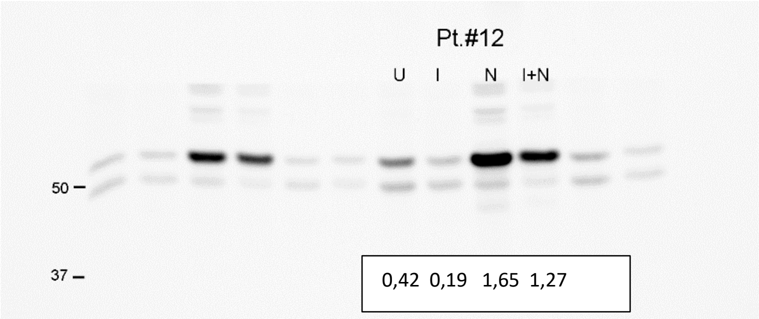

Pt.#13

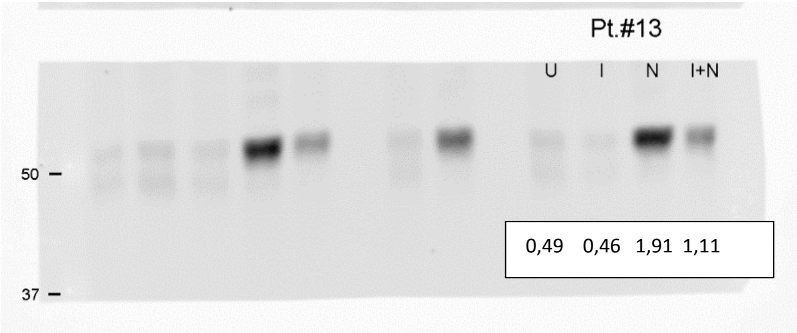

Pt.#14

c-Myc

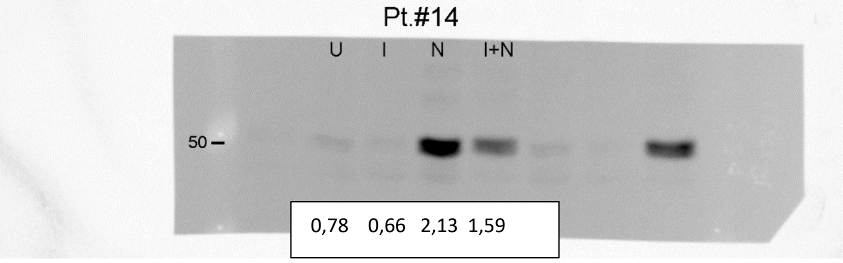

Actin

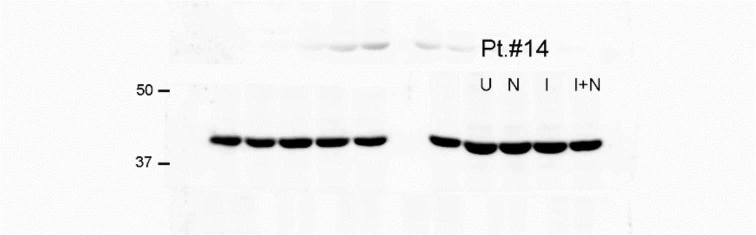

Pt.#16

c-Myc

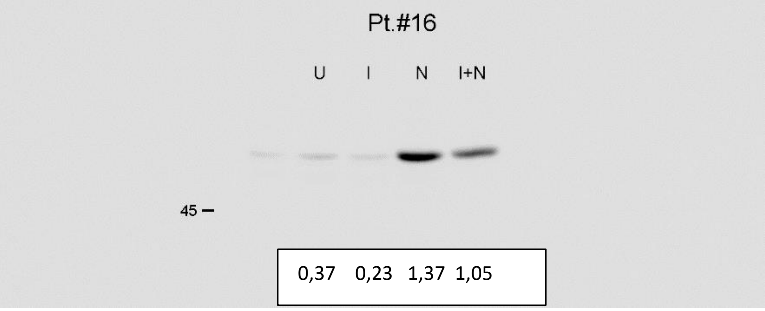

Actin

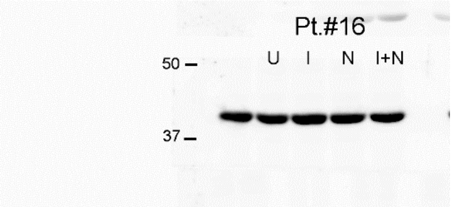

Original blot for Figure 3C

Pt.#12

P-p53 (Ser15)

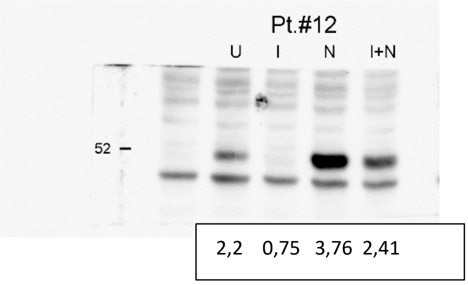

P-p53 (Ser392)

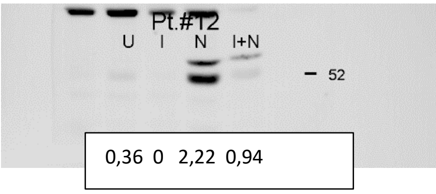

0,36 0 2,22 0,94

PUMA

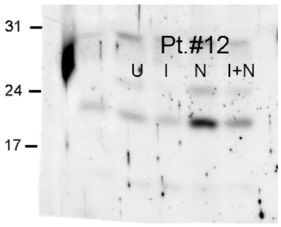

0,29 0,22 0,41 0,24

Actin

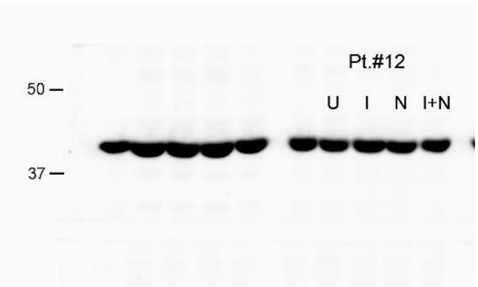

Pt.#16

P-p53 (Ser15)

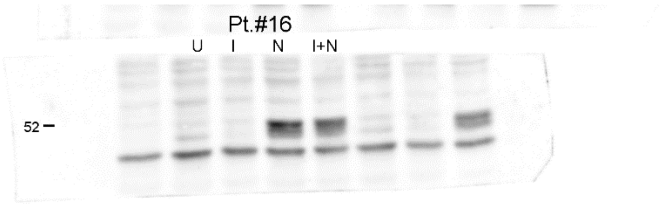

1,05 0,62 2,34 1,98

P-p53 (Ser392)

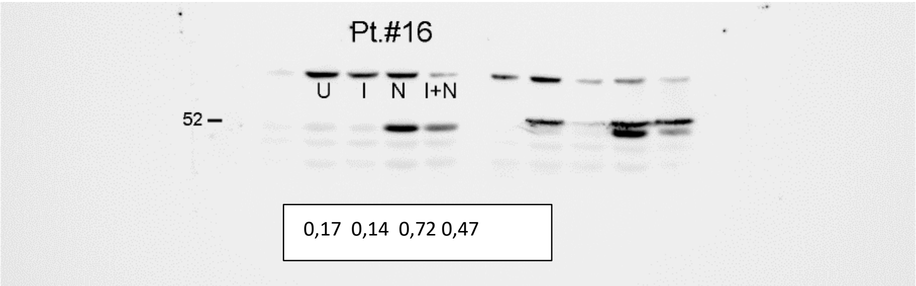

0,17 0,14 0,72 0,47

PUMA

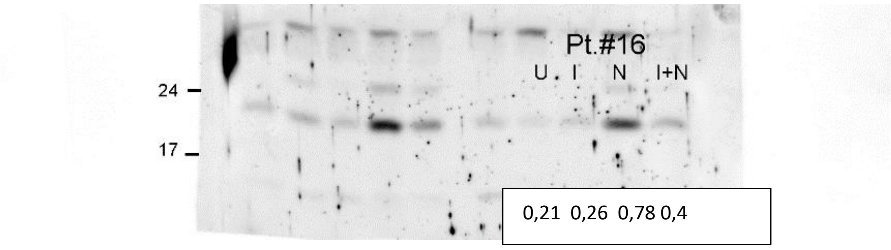

Actin

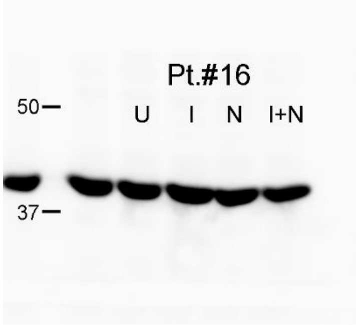

Original bot for Figure 4B

Pt.#12

Pro-Caspase 3

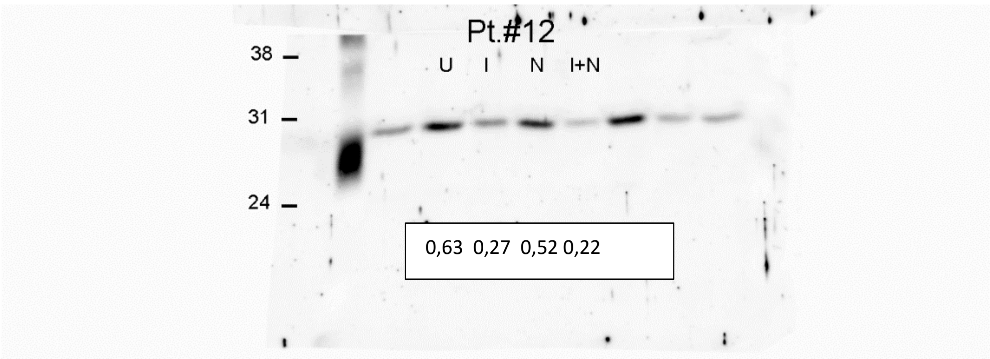

Pro-Caspase 9

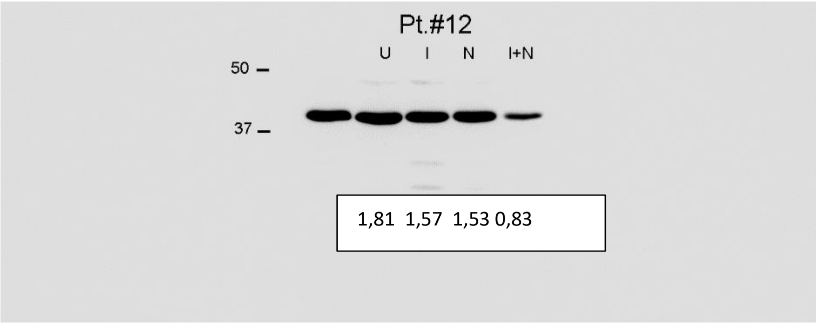

Actin

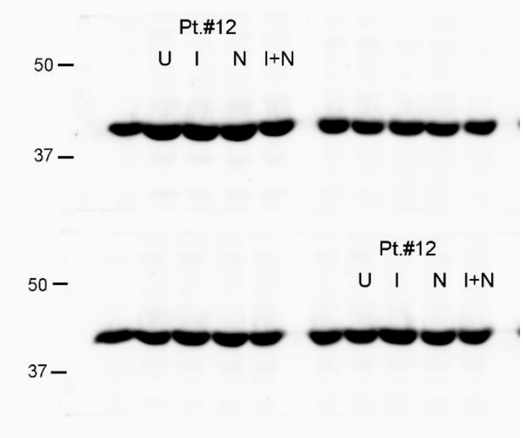

Pt.#16

Pro-Caspase 3

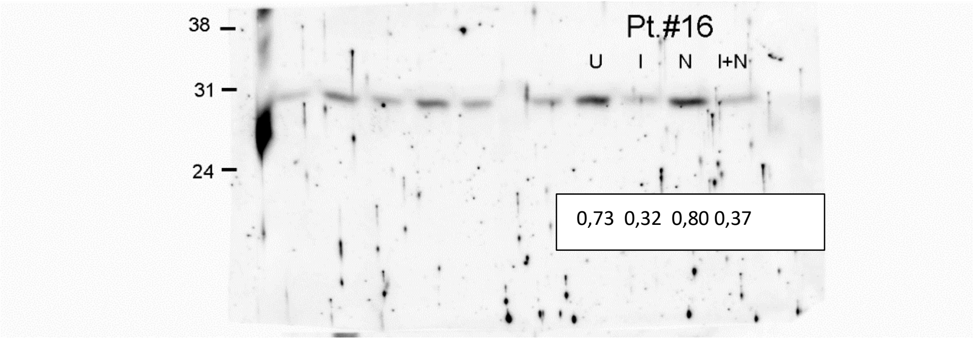

Pro-Caspase 9

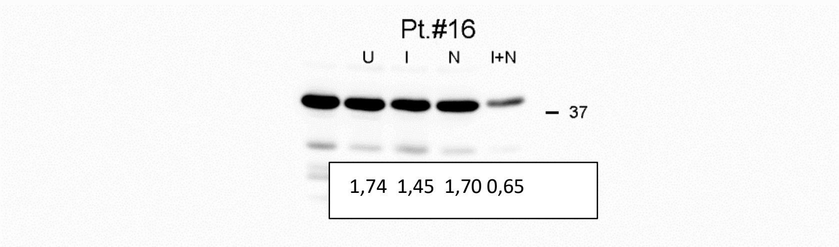

Actin

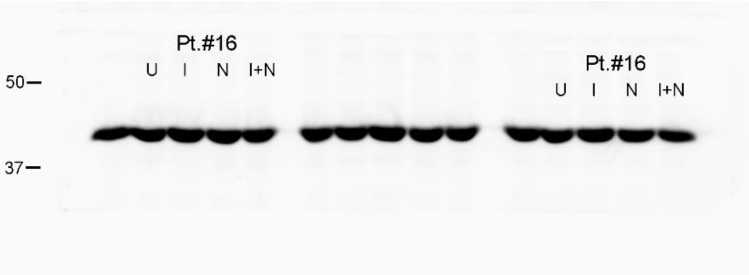

Original bot for Figure 4C

Pt.#12

P-BAD

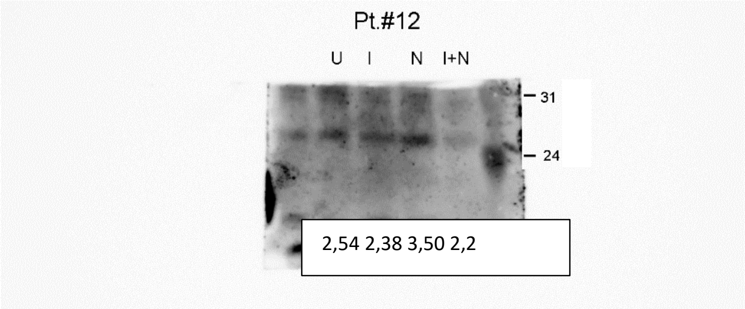

BAD

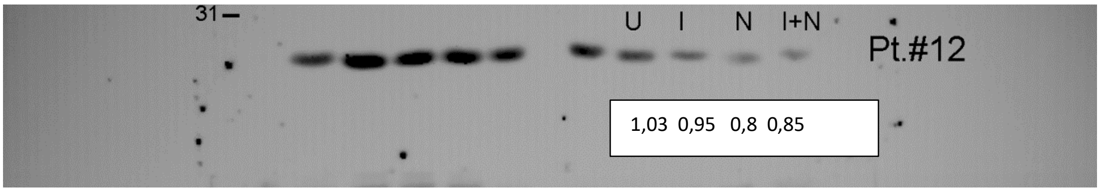

BAX

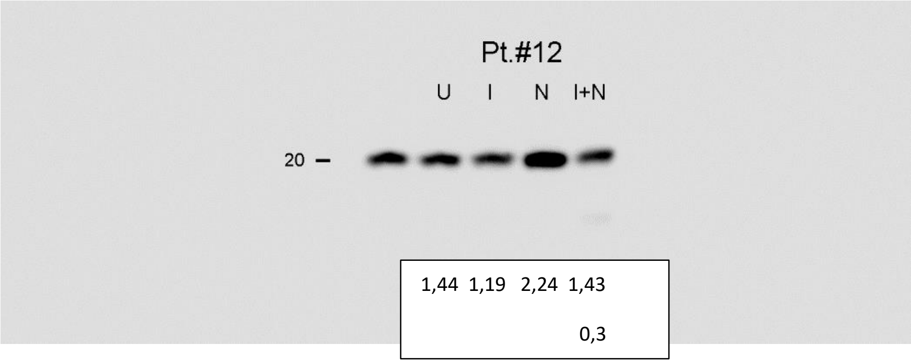

P-Akt

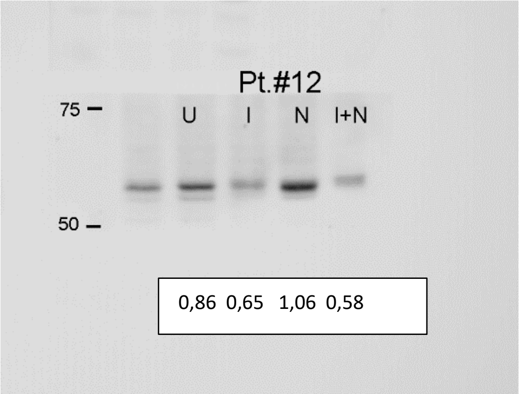

Akt

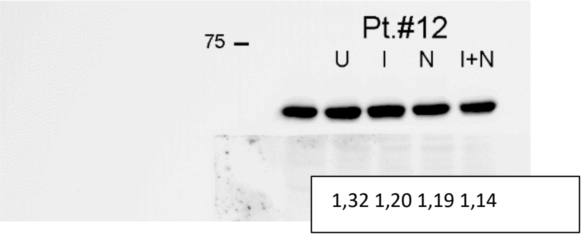

1,32 1,20 1,19 1,14

Actin

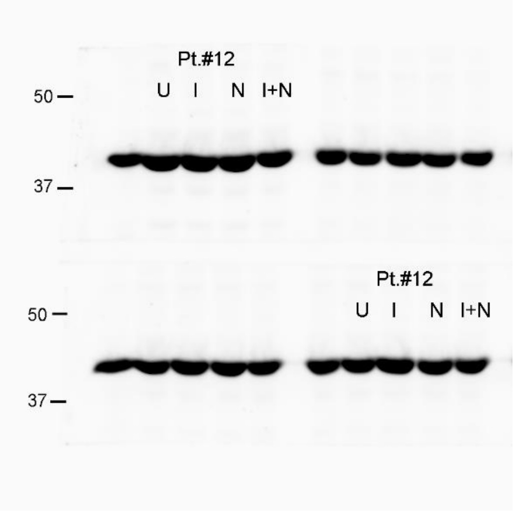

Pt.#16

P-BAD

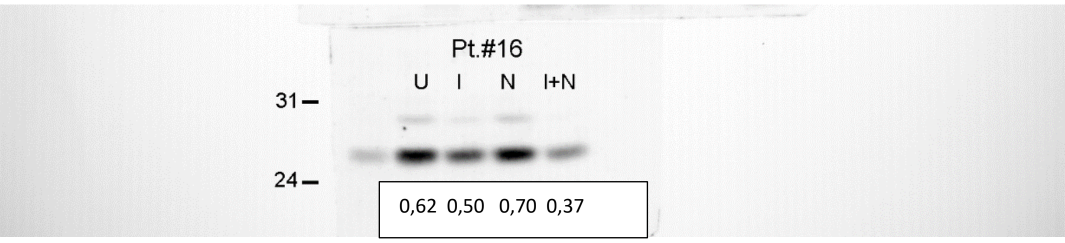

0,62 0,50 0,70 0,37

BAD

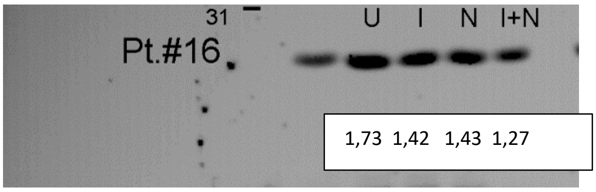

1,73 1,42 1,43 1,27

BAX

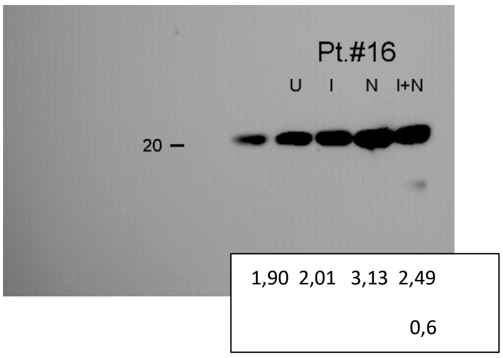

1,90 2,01 3,13 2,49

0,6

P-Akt

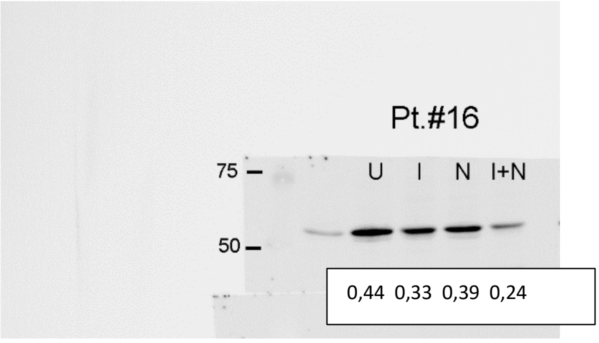

Akt

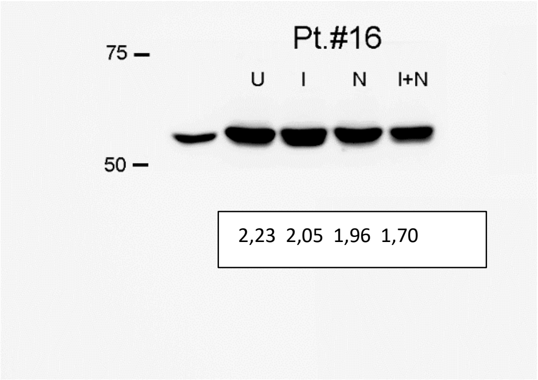

Actin

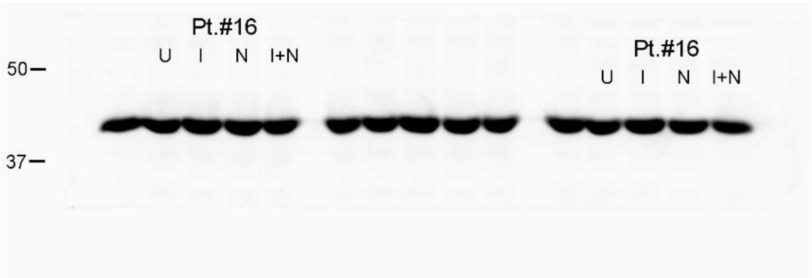

Supplement: Supplementary file 1 [file curroncol-28-00223-s001.zip › curroncol-1272878-supplementary.pdf]
